# Factors associated with adverse pregnancy outcome in Debre Tabor town, Northwest Ethiopia: a case control study

**DOI:** 10.1186/s13104-018-3932-2

**Published:** 2018-11-19

**Authors:** Abraham Sahilemichael Kebede, Achenef Asmamaw Muche, Amelework Getinet Alene

**Affiliations:** 1 Pan Africa University Life and Earth Sciences Institute (PAULESI), University of Ibadan, Ibadan, Nigeria; 2 1000 Days Plus Project, Department of Reproductive and Health Service Management, Addis Ababa University, Addis Ababa, Ethiopia; 3 Department of Epidemiology and Biostatistics, Institute of Public Health, University of Gondar, Gondar, Ethiopia; 4 Amhara Public Health Institute, Public Health Emergency Management Directorate, Bahirdar, Ethiopia

**Keywords:** Adverse pregnancy outcome, Stillbirth, Miscarriage, Debre Tabor town, Northwest Ethiopia, Case control

## Abstract

**Objective:**

The aim of this study was to assess the socioeconomic and demographic factors on adverse pregnancy outcomes.

**Result:**

The mean age of cases was 42.2 (± 13.26) years and the mean age of controls was 34.5 (± 12.23) years. Advanced maternal age, low educational status, and early sexual debut showed a significant association with an adverse pregnancy outcome. Mothers in the age group 35–44 years, AOR 2.54 (95% CI 1.27, 5.06), 35–44 years, AOR 2.79 (95% CI 1.27, 6.16) and Mothers with age 55 years and above AOR 4.18 (95% CI 1.73, 9.13) were more likely to have an adverse pregnancy outcome compared to mothers in the age group ≤ 24 years. The low educational status was also found to have an implication on adverse pregnancy outcome. Those mothers with no formal education were two times more likely to develop adverse pregnancy outcome AOR 2.15 (95% CI 1.41, 2.81) and those in primary education AOR 1.6 (95% CI 1.06, 4.6) times more likely compared to those in higher education.

## Introduction

According to World Health Organization report everyday more than 830 women die from pregnancy and or childbirth related complications around the globe [1]. In 2015 alone, roughly over 303,000 women died during and following pregnancy and childbirth [2].

Since the implementation of the MDGs, countries have showed remarkable progress in reducing unacceptably high maternal mortality. Published studies across countries have revealed that levels of maternal mortality halved since 1990 [1, 3, 4]. Despite the reduction of maternal mortality across the world MMR still continues to be the major index of the widening discrepancy in the level of care and the outcome of reproductive health between the advanced and developing countries. Over 99% of these deaths occurs in low-resource settings, and most could have been prevented [1].

Globally, the adverse pregnancy outcome such as stillbirths, miscarriage, abortions and preterm births are being used as a maternal health indices [5]. More than 60% of preterm births take place in south Asia and sub-Saharan Africa [6]. Similarly, abortions and stillbirths are common adverse pregnancy outcomes that contribute substantially to poor maternal health outcome. Among an estimated 210 million pregnancies, 75 million end in abortion or stillbirth [7, 8].

Study from rural India found 19.8% preterm births, 1.7% abortions and 0.9% stillbirths. A similar study done in rural Uganda found 7.2% abortion and 1.3% in stillbirth [9–11]. A study conducted in Gondar University Hospital have also showed 14.3% of preterm and 7.1% of stillbirths [12, 13].

Evidence has also shown that adverse pregnancy outcomes had a strong and consistent association with biological, social and environmental factors. Many studies have found that socioeconomic status, income inequality and demographic factors are correlate with pregnancy outcomes. Also, a variety of social factors like maternal education, marital status and pregnancy intention have been linked to adverse pregnancy outcomes [14].

Older maternal age was associated with a higher risk of adverse pregnancy outcome [15]. Women who had primary education or above were less likely to have had adverse pregnancy outcomes compared to those with no education [13, 16].

The overall aim of this study was to assess the association between different sociodemographic characteristics and adverse pregnancy outcomes.

## Main text

### Study design, period and setting

Community based Unmatched case control study was conducted from January to June 2015 in Debre Tabor town. The town is located 667 kms away from Addis Ababa the capital of Ethiopia. According to the 2010 Central Statistical Agency (CSA) report the population of Debre Tabor town is estimated to be 78,703 (37,682 males and 41,021 females) [17]. The town is divided into 4 administrative kebeles (the smallest administration unit in Ethiopia). Study participants, both cases and controls were recruited at household level.

### Sample size

The sample size was calculated using a two population proportion formula to determine the number of participants. The proportion of having no formal education, being unemployed and unwanted pregnancy was used to determine the sample size. Unwanted pregnancy in the outcome induced abortion brought a higher sample size among other computed explanatory variables. The following parameter were used: 95% confidence level, 80% power and proportion of women who had a history of abortion among unwanted pregnancies from a study done in southern Ethiopia 75.5% and the odds of induced abortion among wanted pregnancy which was found to be 0.44 were taken into consideration to calculate the minimum sample size [18]. The final calculated sample size with 10% contingency was 620, equally shared among cases and controls (n1 = n2 = 310).

### Data collection and management

Data collectors were trained and employed for the data enumeration. The standard questionnaire to collect the data were designed in English and translated to the local language, Amharic, by a language expert and transcribed back to English to check for consistency. The interview was held in a secluded room after the study participant consented to participate in the study. During the data collection period on spot checking and monitoring was done.

### Outcome variable

History of adverse pregnancy outcomes (preterm, miscarriage, stillbirth and abortion), from the recent pregnancy history was recorded using a detailed interviewer questionnaire. Outcome variable ascertainment was self-report by the study participants. In this study, the Adverse pregnancy outcome was defined as mothers experienced at least one type of the following (preterm, miscarriage, stillbirth and/or abortion) was considered as a case and controls were women who never experienced one of the above situation.

*Stillbirth* is defined as the death of the fetus in the uterus before birth at or after 28-week gestational age [19].

*Preterm* all births before 37 gestational weeks or fewer than 259 days since the first day of a woman’s last menstrual period [19].

*Miscarriage* spontaneous fetal loss before 22 week of gestation [20].

*Abortion* is the termination or initiation of termination of pregnancy before reaching viability (before 28 weeks of gestation or less than 1 kg fetal weight) [21].

### Statistical analysis

STATA version 13 was used to analyze the finding of this study. Descriptive statistics and bivariate analysis was done. Further multiple logistic regression analysis was done to eliminate for potential confounding effect of different socioeconomic and demographic variables.

### Result

This study included 310 cases and 310 controls. The mean age of cases was 42.2 (± 13.26) years and mean age of controls 34.5 (± 12.23) years. Seventy percent of cases and 65.2% of controls were unemployed. The majority 56% of the cases and more than 55% of the controls had no formal education; while only 2% of cases and 4.5% of the controls attended higher education. The majority of the cases and controls 32.3% and 48% respectively were in a relationship for less than 5 years. The different socio-demographic and economic characteristics are given in Table 1.

**Table 1:** Sociodemographic characteristics of cases and controls among women in child bearing age group, Debre Tabor town, Northwest Ethiopia

Variables	Category	Cases, n = 310 (%)	Control, n = 310 (%)	Total, n = 620 (%)	*P* value
Maternal age	≤ 24	22 (7.1)	52 (16.8)	74 (12)	0.00
	25–34	79 (25.5)	139 (44.8)	218 (35)	
	35–44	80 (25.8)	60 (19.35)	140 (22.6)	
	45–54	68 (22)	38 (12.3)	106 (17.1)	
	≥ 55	61 (21.9)	21 (6.8)	82 (13.2)	
Residence	Rural	112 (36.1)	83 (26.8)	195 (31.5)	0.012
	Urban	198 (63.9)	227 (73.2)	425 (68.6)	
Religion	Christian	265 (85.5)	258 (83.2)	523 (84.4)	0.439
	Muslim	45 (14.5)	52 (16.8)	97 (15.6)	
Ethnicity	Amhara	292 (94.2)	294 (94.84)	586 (94.5)	0.586
	Oromo	5 (1.6)	7 (2.3)	12 (1.9)	
	Tigre	13 (4.2)	9 (3)	22 (3.5)	
Educational status	No formal education	174 (56)	171 (55.2)	345 (55.6%)	0.125
	Primary education	94 (30.3)	79 (25.5)	173 (28%)	
	Secondary education	36 (12)	46 (14.8)	82 (13.2%)	
	Higher education	6 (2)	14 (4.5)	20 (3.2)	
Employment status	Unemployed	218 (70.3)	202 (65.2)	420 (67.7)	0.169
	Employed	92 (30)	108 (34.8)	200 (32.3)	
Family Income categorized	≤ 560	99 (32)	70 (22.6)	169 (27.3)	0.03
	561–900	83 (26)	80 (25.8)	163 (26.3)	
	901–1700	65 (21)	87 (28)	152 (24.5)	
	≥ 1701	63 (20)	73 (23.6)	136 (22)	
Age at time first sex	≤ 14	63 (20)	20 (6.5)	83 (13.4)	0.00
	15–17	87 (28)	85 (27.4)	172 (28)	
	≥ 18	160 (21.6)	205 (66)	365 (58.9)	
Previous history of marriage	No	10 (3.2)	16 (5.2)	26 (4.2)	0.229
	Yes	300 (96.8)	294 (95)	594 (95.8)	
Duration of live in marriage	≤ 5	100 (32.3)	149 (48)	249 (40.2)	0.00
	6–10	72 (23)	75 (24.2)	147 (23.7)	
	11–15	46 (14.8)	36 (11.2)	82 (13)	
	≥ 16	92 (30)	50 (16)	142 (23)	
Age difference between the partner at first intercourse	Equal	40 (13)	69 (22.3)	109 (17.6)	0.00
	< 5 years	56 (18)	90 (29)	146 (23.6)	
	≥ 5 years	214 (69)	151 (48.7)	365 (59)	
Type of marriage	Monogamous	239 (77)	242 (78)	481 (77.6)	0.773
	Polygamous	71 (23)	68 (22)	139 (22.4)	

*P* value of pearson’s chi square test to compare cases and controls

In our study, urban residents were majority in both groups. Sixty-three percent of cases and 73.2% of controls were urban dwellers. The majority of study participants were Christian in religion in both groups. And also the majority of the study participants in both the cases and controls were from the Amhara ethnic group. Both in the case and control group’s majority, 96.8% and 95% of the cases and controls had previous history of marriage. Sexual debut was significantly different between the cases and controls. The majority of the cases 28% initiated sex at the age between 15 and 17 years while 66% of controls initiate sex after age of 18 years and above.

Maternal age, residence of the respondent, marital status, educational status, family income, age at first sex, age difference with partner at first sexual intercourse and duration of relationship found to have a significant association on the bivariate analysis; these variables further fitted into a multivariate logistic regression model.

Advanced maternal age was found to be significantly associated with higher odds of adverse pregnancy outcome. Mothers in the age group 35–44 years of age were 2.5 times more likely to counter an adverse pregnancy outcome AOR 2.54 (95% CI 1.27, 5.06) similarly mothers in the age 45–54 years were significantly associated with adverse pregnancy outcome AOR 2.79 (95% CI 1.27, 6.16).

Educational status also showed a significant association with adverse pregnancy outcome. Those mothers with no formal education were 2 times more likely to develop an adverse pregnancy outcome AOR 2.15 (95% CI 1.41, 2.81) and mothers with primary education were 1.6 times more likely to have an adverse pregnancy outcome compared to those in higher education AOR 1.6 (95% CI 1.06, 4.6).

Women living in rural area were twice as likely to be at risk of adverse pregnancy outcome compared to women living in urban AOR 1.51 (95% CI 1.03, 2.21). The odds of adverse pregnancy outcome were higher among women with ≥ 5 years age difference with their partner at first sex compared to those with zero age difference AOR 1.74 (95% CI 1.07, 2.83). Early debut for sex was also associated with adverse pregnancy outcome among mothers. Odds of adverse pregnancy outcome were two times higher among women who started sex less than 14 years age compared to those who started sex ≥ 18 years of age AOR 2.29 (95% CI 1.25, 4.23) (Table 2).

**Table 2:** The univariate and multivariate analysis of the determinants of adverse pregnancy outcome for different explanatory variables categories, Debre Tabor town, Northwest Ethiopia

Variables	Category	Cases, n = 310 (%)	Control, n = 310 (%)	COR (95% CI)	AOR (95% CI)
Maternal age	≤ 24	22 (7.1)	52 (16.8)	1	1
	25–34	79 (25.5)	139 (44.8)	1.34 (0.76, 2.37)	1.15 (0.63, 2.1)
	35–44	80 (25.8)	60 (19.35)	3.15 (1.73, 5.74)*	2.54 (1.27, 5.06)*
	45–54	68 (22)	38 (12.3)	4.23 (2.24, 7.99)*	2.79 (1.27, 6.16)*
	≥ 55	61 (21.9)	21 (6.8)	6.86 (3.39, 13.87)*	4.18 (1.73, 9.13)*
Residence	Rural	112 (36.1)	83 (26.8)	1.55 (1.09, 2.18)*	1.51 (1.03, 2.21)*
	Urban	198 (63.9)	227 (73.2)	1	1
Marital status	Married	199 (64.2)	235 (75.8)	1	1
	Divorced	54 (17.4)	37 (12)	1.72 (1.08, 2.73)*	1.08 (0.64, 1.83)
	Widowed	40 (13)	17 (5.5)	2.78 (1.53, 5.05)*	1.02 (0.5, 2.07)
	Separated	17 (5.5)	21 (6.8)	0.96 (0.49, 1.86)	0.95 (0.46, 1.93)
Educational status	No formal education	174 (56)	171 (55.2)	2.37 (0.89, 6.32)	2.15 (1.41, 2.81)*
	Primary education	94 (30.3)	79 (25.5)	2.77 (1.01, 7.56)*	1.6 (1.06, 4.6)*
	Secondary education	36 (12)	46 (14.8)	1.83 (0.64, 5.22)	1.45 (0.48, 1.93)
	Higher education	6 (2)	14 (4.5)	1	1
Job status	Unemployed	218 (70.3)	202 (65.2)	1.23 (0.9, 1.77)	
	Employed	92 (30)	108 (34.8)	1	
Family income categorized	≤ 560	99 (32)	70 (22.6)	1.6 (1.03, 2.58)*	1.23 (0.72, 2.11)
	561–900	83 (26)	80 (25.8)	1.2 (0.76, 1.89)	1.14 (0.68, 1.94)
	901–1700	65 (21)	87 (28)	0.86 (0.54, 1.38)	0.93 (0.56, 1.57)
	≥ 1701	63 (20)	73 (23.6)	1	1
Age at time first sex	≤ 14	63 (20)	20 (6.5)	4.03 (2.34, 6.95)*	2.29 (1.25, 4.23)*
	15–17	87 (28)	85 (27.4)	1.31 (0.911, 1.88)	1.32 (0.89, 2.19)
	≥18	160 (21.6)	205 (66)	1	1
Duration of relationship	≤ 5	100 (32.3)	149 (48)	1	1
	6–10	72 (23)	75 (24.2)	1.43 (0.95, 2.16)	1.3 (0.86, 2.19)
	11–15	46 (14.8)	36 (11.2)	1.90 (1.14, 3.15)*	1.04 (0.57, 1.92)
	≥ 16	92 (30)	50 (16)	2.74 (1.79, 4.2)*	1.24 (0.68, 2.28)
Previous history of marriage	Yes	300 (96.8)	294 (95)	1.63 (0.73, 3.65)	
	No	10 (3.2)	16 (5.2)	1	
Age difference between the partner at first intercourse	No difference	40 (13)	69 (22.3)	1	1
	< 5 years	56 (18)	90 (29)	1.07 (0.64, 1.79)	1.07 (0.6, 1.89)
	≥ 5 years	214 (69)	151 (48.7)	2.44 (1.57, 3.80)*	1.74 (1.07, 2.83)*
Type of marriage	Monogamous	239 (77)	242 (78)	Ref	
	Polygamous	71 (23)	68 (22)	1.05 (0.72, 1.54)	

1 = Reference

*P < 0.05

**P < 0.01

### Discussion

This study was conducted to identify socioeconomic and demographic factors associated with adverse pregnancy outcome in Debre Tabor town, Northwest Ethiopia.

In our study, advanced maternal age has showed a strong association with adverse pregnancy outcome. In line with this finding several studies done in different areas have revealed similar findings that older age was associated with higher odds of stillbirth [13, 22, 23]. Study done in Denmark on a nationally representative survey, which have showed advanced maternal age is the risk of adverse pregnancy outcome [20]. This might be due to the aging process in the ovaries [24].

The finding also revealed that as the age of the mother increases the association with an adverse pregnancy outcome becomes stronger. While a multicounty analysis of the effect of maternal age on adverse pregnancy outcome showed a higher risk among adolescent mothers [25]. This difference might be attributed to the difference in measurement as the study was comparing risk of adverse outcome among early and late adolescent mothers. This can be explained because early pregnancy is highly associated with immature physiologic maternal change which can lead to a higher risk [26, 27].

Mothers with low educational status were also at risk of developing an adverse pregnancy outcome compared to the mother with a higher education. This was in line with the study done in the Amhara region of Ethiopia, which found low education was associated with higher odds of adverse pregnancy outcome [13]. As having lower educational status was accompanied by low maternal and neonatal service utilization. A study has also shown a high tendency of using maternity waiting home among educated women [22]. This finding was opposed by the studies which indicated high rates of abortions among those with higher education [9, 11].

On the other side early sexual debut was significantly associated with adverse pregnancy outcome AOR 2.29 (95% CI 1.25, 4.23). This finding was similar with a study done in Australia, which revealed early sexual initiation associated with adverse pregnancy outcome [28].

### Conclusions

Advanced maternal age, low educational status, early sexual debut and age difference with a partner of 5 years and above during the first sexual intercourse, have showed increased the odds of adverse pregnancy outcome.

### Limitation of the study

- Determination of the outcome variable totally depended on the study participant memory and awareness on the issue.
- This study is limited to socioeconomic and demographic factors.

## Abbreviations

AOR: adjusted odds ratio
CI: confidence interval
MDGs: Millennium Developmental Goals
MMR: maternal mortality ratio
LMIC: lower and middle income countries
EDHS: Ethiopian Demographic and Health Survey
WHO: World Health Organizations
